# Mutations in *gfpt1* and *skiv2l2* Cause Distinct Stage-Specific Defects in Larval Melanocyte Regeneration in Zebrafish

**DOI:** 10.1371/journal.pgen.0030088

**Published:** 2007-06-01

**Authors:** Chao-Tsung Yang, Anna E Hindes, Keith A Hultman, Stephen L Johnson

**Affiliations:** Department of Genetics, Washington University School of Medicine, Saint Louis, Missouri, United States; Stanford University School of Medicine, United States of America

## Abstract

The establishment of a single cell type regeneration paradigm in the zebrafish provides an opportunity to investigate the genetic mechanisms specific to regeneration processes. We previously demonstrated that regeneration melanocytes arise from cell division of the otherwise quiescent melanocyte precursors following larval melanocyte ablation with a small molecule, MoTP. The ease of ablating melanocytes by MoTP allows us to conduct a forward genetic screen for mechanisms specific to regeneration from such precursors or stem cells. Here, we reported the identification of two mutants, *eartha* ^j23e1^ and *julie* ^j24e1^ from a melanocyte ablation screen. Both mutants develop normal larval melanocytes, but upon melanocyte ablation, each mutation results in a distinct stage-specific defect in melanocyte regeneration. Positional cloning reveals that the *eartha* ^j23e1^ mutation is a nonsense mutation in *gfpt1 (glutamine:fructose-6-phosphate aminotransferase 1),* the rate-limiting enzyme in glucosamine-6-phosphate biosynthesis. Our analyses reveal that a mutation in *gfpt1* specifically affects melanocyte differentiation (marked by melanin production) at a late stage during regeneration and that *gfpt1* acts cell autonomously in melanocytes to promote ontogenetic melanocyte darkening. We identified that the *julie* ^j24e1^ mutation is a splice-site mutation in *skiv2l2 (superkiller viralicidic activity 2-like 2),* a predicted DEAD-box RNA helicase. Our in situ analysis reveals that the mutation in *skiv2l2* causes defects in cell proliferation, suggesting that *skiv2l2* plays a role in regulating melanoblast proliferation during early stages of melanocyte regeneration. This finding is consistent with previously described role for cell division during larval melanocyte regeneration. The analyses of these mutants reveal their stage-specific roles in melanocyte regeneration. Interestingly, these mutants identify regeneration-specific functions not only in early stages of the regeneration process, but also in late stages of differentiation of the regenerating melanocyte. We suggest that mechanisms of regeneration identified in this mutant screen may reveal fundamental differences between the mechanisms that establish differentiated cells during embryogenesis, and those involved in larval or adult growth.

## Author Summary

Programs of ontogenetic development and regeneration share many components. Differences in genetic requirements between regeneration and development may identify mechanisms specific to the stem cells that maintain cell populations in postembryonic stages, or identify other regeneration-specific functions. Here, we utilize a forward genetic approach that takes advantage of single cell type ablation and regeneration to isolate mechanisms specific to regeneration of the zebrafish melanocyte. Upon chemical ablation of melanocytes, zebrafish larvae reconstitute their larval pigment pattern from undifferentiated precursors or stem cells. We isolated two zebrafish mutants that develop embryonic melanocytes normally but fail to regenerate their melanocytes upon ablation. This phenotype suggests the regeneration-specific roles of the mutated genes. We further identified the mutations in *gfpt1* and *skiv2l2* and show their stage-specific roles in melanocyte regeneration. Interestingly, these mutants identify regeneration-specific functions not only in early stages of the regeneration process *(skiv2l2),* but also in late stages of differentiation of the regenerating melanocyte *(gfpt1)*. We suggest that mechanisms of regeneration identified in this mutant screen may reveal fundamental differences between the mechanisms that establish differentiated cells during embryogenesis and those involved in larval or adult growth.

## Introduction

Regeneration and turnover of cells and tissues are essential in many vertebrates, including humans. Tissue homeostasis and regeneration have often been demonstrated to act through recruiting quiescent undifferentiated precursors or stem cells to re-enter developmental programs reminiscent of embryonic mechanisms. The mechanisms regulating these processes are critical for maintaining normal physiology. For instance, uncontrolled growth of undifferentiated precursors or stem cells has been suggested to cause many types of cancers, including leukemia and skin cancer [1–5]. However, little is known about the genes and pathways that specifically regulate these precursors or stem cells. The fact that the differentiation and physiological states of adult animals are very distinct from embryos raises the possible existence of pathways or mechanisms specific to regeneration or to those mechanisms that regulate the development of these undifferentiated cells. Such mechanisms may play roles in establishing the precursors or stem cells during embryogenesis, maintaining them throughout development, or recruiting them at the adult stages for growth, regeneration, or tissue homeostasis. Understanding the mechanisms specific to regeneration or homeostasis will provide insight into the regulation of stem cells, which may lead to the development of therapies for many human diseases.

Regeneration studies are classically performed by amputating a mass of continuous tissues, such as a limb or a portion of an organ, and investigating how organisms coordinately regenerate multiple types of cells or tissues [6,7]. Recently, some attention has been focused on single cell type ablation and subsequent regeneration [8–11]. This approach has two advantages. First, focusing on a single cell type reduces the complexity of the regeneration process, and second, it provides an opportunity to isolate the mechanisms that control adult stem cells from other biological mechanisms that may be occurring during the normal development of that cell type. Additionally, it may also provide an opportunity to understand the cause or cure for human disease resulting from single cell type deficits, such as loss of β cells in autoimmune type I diabetes [12]. Several methods that ablate single cell types have been reported in various vertebrates, including genetic approaches, and use of lasers or chemicals [10,13–16]. For example, Lamotte et al. [13] used transgenic methods to introduce tissue-specific expression of a toxin to ablate mouse pancreatic β cells. Other examples include exposure to a dermatology laser and incubation with a small molecule, 4-(4-morpholinobutylthio)phenol (MoTP), each of which has been shown to specifically ablate zebrafish larval melanocytes [9,17].

The zebrafish larval melanocyte is an attractive cell type for regeneration studies. Mature larval melanocytes can be easily followed by the presence of black pigment; they are dispensable for fish viability in the laboratory, and multiple mutations have been generated that affect different aspects of melanocyte development at different stages of the fish life cycle [18–20]. Most importantly, after the establishment of the larval melanocyte pattern during the first three days of fish development, the number of larval melanocytes remains relatively constant throughout the following two weeks of development with little or no turnover. This period of stable melanocyte pattern provides an opportunity to study melanocyte regeneration once ablated. We previously reported that a small molecule MoTP specifically ablates melanocytes or melanoblasts in zebrafish larvae. The melanocytotoxicity of MoTP is mediated via tyrosinase activity, presumably to convert MoTP into cytotoxic quinone species. Following melanocyte ablation by MoTP, regeneration melanocytes arise from cell division of the otherwise quiescent melanocyte precursors that we suggest are equivalent to adult stem cells [9]. The ease of ablating larval melanocytes by MoTP allows us to conduct a forward genetic screen for mechanisms specific to regeneration from such precursors or stem cells.

Here, we report a melanocyte ablation screen in zebrafish aimed at isolating mutants that fail to regenerate melanocytes. In this mutant screen, we identified two mutants, *eartha* ^j23e1^ and *julie* ^j24e1^. These mutants have normal larval melanocyte development, but upon subsequent melanocyte ablation by MoTP, each mutant largely fails to regenerate their melanocytes. Our analyses revealed that *eartha* ^j23e1^ specifically affects melanocyte differentiation (marked by melanin production) at a late stage during regeneration. Positional cloning reveals that the *eartha* ^j23e1^ mutation is a nonsense mutation in *gfpt1 (glutamine:fructose-6-phosphate aminotransferase 1),* the rate-limiting enzyme that catalyzes the formation of glucosamine-6-phosphate in the hexosamine biosynthesis pathway [21–23]. The major end product of this pathway is UDP-N-acetylglucosamine (UDP-GlcNAc), which is one of the building blocks for glycosyl side chains for many glycoproteins and proteoglycans. Although our analyses on chondrocyte development in *eartha* ^j23e1^ mutants indicate that mutation in *gfpt1* affects the extracellular matrix (ECM) formation of cartilage, our chimera analysis reveals that *gfpt1* acts cell autonomously in melanocytes to promote ontogenetic melanocyte darkening. These findings allow us to infer that *gfpt1* acts cell autonomously in melanocytes to promote melanocyte differentiation from *dopachrome tautomerase* ^+^ *(dct* ^+^); melanin^−^ to melanin^+^ stage during melanocyte regeneration.

We identified the *julie* ^j24e1^ mutation as a splice-site mutation in *superkiller viralicidic activity 2-like 2 (skiv2l2),* which is predicted as a DEAD-box RNA helicase and is proposed to function in regulating various aspects of RNA metabolism in the cells [24]. Our in situ analysis reveals that *skiv2l2* plays an important role in cell proliferation at postembryonic stages, consistent with the role for cell division during melanocyte regeneration we previously described [9].

## Results

### Mutations Specific to Larval Melanocyte Regeneration

**Figure 1:**
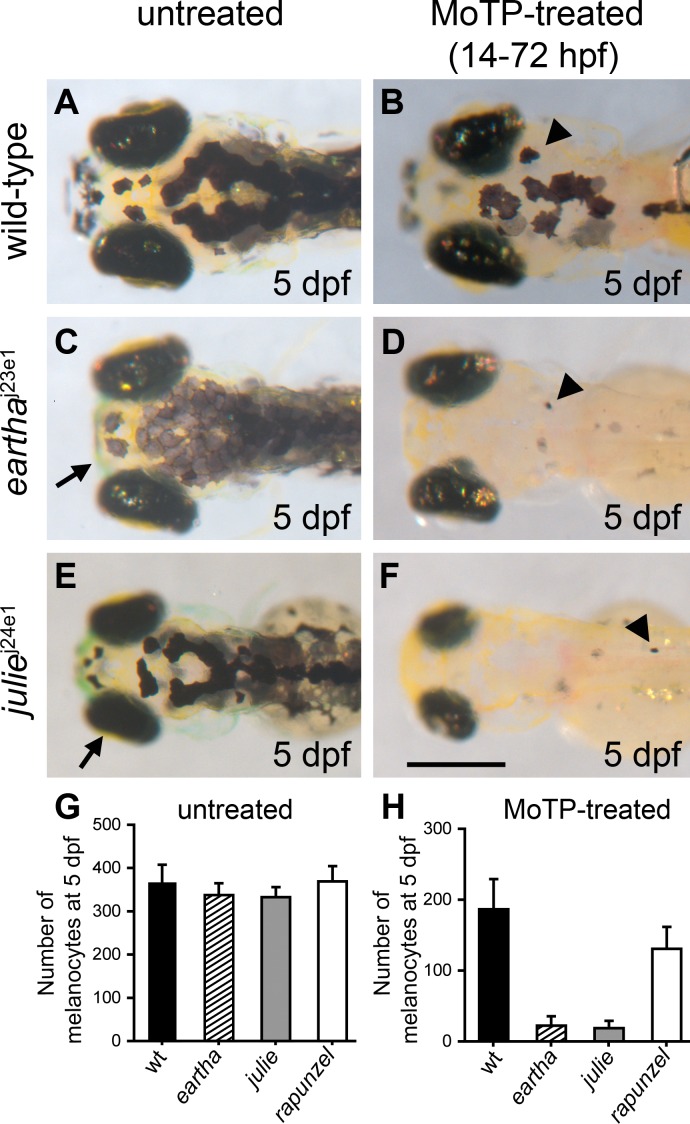
Mutations Specific to Melanocyte Regeneration (A–F) *eartha* ^j23e1^ (C) and *julie* ^j24e1^ mutants (E) both develop larval melanocyte patterns nearly identical to wild-type larvae (A), although melanocytes in *eartha* ^j23e1^ mutants appear to have lighter pigmentation than that in the wild-type larvae. Upon larval melanocyte ablation by MoTP (14–72 hpf), wild-type larvae (B) regenerate many melanocytes at 5 dpf (arrowhead in [B]), while *eartha* ^j23e1^ (arrowhead in [D]) and *julie* ^j24e1^ (arrowhead in [F]) mutants largely fail to regenerate melanocytes. Additional phenotypes are indicated by an arrow in (C) marking a shortened pharyngeal skeleton in *eartha* ^j23e1^ mutants and by an arrow in (E) indicating small eyes and head in *julie* ^j24e1^ mutants. (G–H) The numbers of melanocytes in the untreated (G) and MoTP-treated (H) wild-type and mutant larvae at 5 dpf. Scale bar: 300 μm.

To understand the genetic mechanisms underlying larval melanocyte regeneration, we conducted a parthenogenesis or early-pressure (EP) screen for mutations that specifically block larval melanocyte regeneration, but allow ontogenetic melanocyte development during embryogenesis. We ablated larval melanocytes by incubating embryos in MoTP from 14 to 72 hours postfertilization (hpf) and scored their melanocyte regeneration at 5–8 days postfertilization (dpf) when wild-type larvae have regenerated many melanocytes (Figure 1A and 1B) [9]. Among the 648 EP clutches generated, 421 clutches had more than ten individuals survive through the early pressure and MoTP incubation procedure for melanocyte regeneration analysis. A total of 29 of these clutches and their *F*_1_ mothers were kept as putative mutants, because each of these EP clutches had at least one individual lacking melanocytes after MoTP washout. In addition to these 421 clutches, seven small clutches (fewer than ten surviving individuals) were also kept since at least one of the surviving individuals showed defects in melanocyte regeneration. In total, we found 36 clutches with possible defects in melanocyte regeneration. We then excluded 12 of these 36 clutches from further characterization because additional phenotypes in ontogenetic melanocyte development were observed in their haploid siblings. Among the remaining 24 possible melanocyte regeneration-specific mutants, we successfully rescreened 15 (62%) of them, either by a second EP screen of the *F*_1_ female or from the EP embryos of *F*_2_ outcross, indicating that we effectively screened approximately 263 (421 × 0.62) haploid genomes. Among these 15 possible mutant lines, only two repeated their melanocyte regeneration phenotypes. This high false positive noise could be due to the frequent developmental defects that were caused by the early pressure procedure. Because these mutagenesis protocols typically identify mutations in targeted loci at frequencies of approximately one per 1,000 haploid genomes [25,26], our result suggests that there may be many more genes that could be found by the further exploitation of this melanocyte ablation screen. We followed our laboratory's cat theme for naming pigment mutants. Because the Catwoman character is regenerated with each new actress that plays the role, we chose to name our two regeneration mutations, *eartha* ^j23e1^ and *julie* ^j24e1^ after Catwoman actresses Eartha Kitt and Julie Newmar.

Both *eartha* ^j23e1^ and *julie* ^j24e1^ mutants develop ontogenetic melanocytes normally (Figure 1C, 1E, and 1G), but following melanocyte ablation by MoTP, *eartha* ^j23e1^ and *julie* ^j24e1^ mutants only regenerate 11.8% and 10.0%, respectively, of the normal complement of regeneration melanocytes at 5 dpf (Figure 1D, 1F, and 1H). Both *eartha* ^j23e1^ and *julie* ^j24e1^ mutations are recessive lethal, with most *eartha* ^j23e1^ mutants dying at approximately 12 dpf, and *julie* ^j24e1^ mutants dying at approximately 7–8 dpf. This raises the possibility that the larval melanocyte regeneration defects observed in *eartha* ^j23e1^ and *julie* ^j24e1^ mutants are the consequences of nonspecific larval-stage lethality rather than specific effects on melanocyte regeneration. To help rule out this possibility, we examined melanocyte regeneration in homozygous mutants for an early larval lethal mutation, *rapunzel* ^c14^ [27]. Although homozygous *rapunzel* ^c14^ larvae show many developmental defects including defects in jaw and fin-fold development (unpublished data), the number of melanocytes in *rapunzel* ^c14^ larvae is indistinguishable from that in wild-type larvae at 3 dpf (Figure 1G). By 5 dpf, almost all *rapunzel* ^c14^ larvae have severe edema and then die at approximately 6 dpf. Upon melanocyte ablation by MoTP (14–72 hpf), we found that *rapunzel* ^c14^ mutants regenerate approximately 70% of the number of the melanocytes that regenerate in wild-type larvae, 7-fold more melanocytes than those that regenerate in *eartha* ^j23e1^ and *julie* ^j24e1^ mutants (Figure 1H). This result rules out a strict correlation between larval-stage lethality and defects in melanocyte regeneration, and also suggests that the regeneration defects observed in *eartha* ^j23e1^ and *julie* ^j24e1^ larvae result from defects specific to regeneration mechanisms.

### *eartha* Mutation Specifically Affects Larval Melanocyte Regeneration after dct^+^ Stage

**Figure 2:**
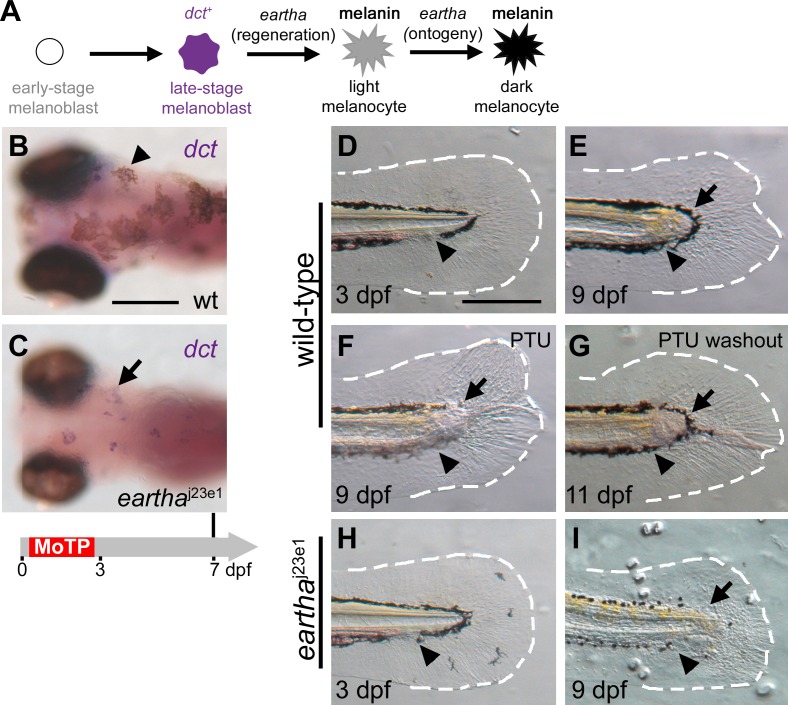
The *eartha* ^j23e1^ Mutation Specifically Affects Melanocyte Regeneration after the *dct* ^+^ Stage but Has No Effect on Larval Tail Regeneration (A) The roles of *eartha* in different stages of melanocyte development in ontogeny and regeneration are presented. (B and C) Larval melanocytes in the wild-type (B) and *eartha* ^j23e1^ (C) larvae were ablated by MoTP treatment from 14 to 72 hpf, and melanocyte regeneration was analyzed at 7 dpf by ISH with *dct* riboprobes for late-stage melanoblasts. At this stage, many melanocytes (melanin^+^) have regenerated in the wild-type larvae (arrowhead in [B]). Although only few pigmented melanocytes appear in *eartha* ^j23e1^ larvae at the same stage, many *dct* ^+^ melanoblasts (melanin^−^, stained purple) are revealed by ISH (arrow in [C]). (D–I) *eartha* ^j23e1^ mutation specifically affects melanocyte regeneration during larval tail regeneration. Arrowheads show amputation planes at 3 dpf prior to amputation (D) and (H) and after regeneration (E–G) and (I). Wild-type larval tails (D) were amputated at 3 dpf. The wound healed in the ensuing two days by closing the injured tip of the notochord and neural tube, followed by the outgrowth of fin fold. By 9 dpf (6 d postamputation), the missing larval tails were reconstituted, including fin-fold tissues (outlined with white-dashed lines) and melanocytes (arrow in [E]). The origins of the re-established tail melanocytes were investigated by incubating larvae in PTU immediately after larval tail amputations (F). PTU prevents melanin synthesis in newly differentiated melanocytes, and thus the pigmented melanocytes that appear in regenerated tails of the PTU-treated larvae pre-existed prior to the tail amputation. At 9 dpf, few melanocytes (arrow in [F]) appear in the newly regenerated tail immediately distal to the amputation plane in the PTU-treated larvae, suggesting that these melanocytes arise from the migration of proximately differentiated melanocytes from the stump (F). Additional lightly pigmented melanocytes appear after PTU washout (arrow in [G]), indicating that these melanocytes are newly differentiated. When similar tail amputations were conducted in *eartha* ^j23e1^ larvae (H and I), the larval tails regenerated identically to wild-type larvae, except that few melanocytes appear in the regenerated tail in the region immediately distal to the amputation plane (arrow in [I]). The white-dashed lines outline the fin folds. Timeline (grey) below (C) indicates the period of MoTP treatment (red) and analysis time (vertical line above the timeline). Scale bars: 250 μm.

The development of *eartha* ^j23e1^ embryos is indistinguishable from wild-type embryos until 3–4 dpf at which stage their melanocytes appear lighter than wild-type larvae (Figure 1C). This observation suggests that *eartha* plays a role in ontogenetic melanocyte darkening (Figure 2A). Additional defects appear after 5 dpf in *eartha* ^j23e1^ larvae, including a defect in jaw development, where the jaw fails to grow anterior beyond the eyes, typically described as the hammerhead phenotype (Figure 1C) [28]. *eartha* ^j23e1^ mutants develop swim bladders normally, but most die by 12 dpf, presumably a consequence of the pharyngeal skeleton defect leading to an inability to feed. Upon melanocyte ablation by MoTP treatment, only 11.8 % of melanocytes regenerate at 5 dpf in *eartha* ^j23e1^ animals, as compared to wild-type larvae (animals examined 2 d postMoTP treatment) (Figure 1B and 1D).

To further understand how the *eartha* ^j23e1^ mutation affects melanocyte regeneration, we examined melanocyte differentiation in *eartha* ^j23e1^ larvae during melanocyte regeneration after MoTP washout. Larval melanocytes in *eartha* ^j23e1^ larvae were ablated by MoTP treatment from 14 to 72 hpf, and regenerated melanocytes were analyzed at 7 dpf by in situ hybridization (ISH) analysis, a time when many melanocytes regenerate in wild-type larvae (Figure 2B). Although *eartha* ^j23e1^ larvae had only very few regenerated melanocytes (melanin^+^), we detected many *dct* ^+^ melanoblasts (melanin^−^) in the *eartha* ^j23e1^ larvae (Figure 2C). The number of these *dct* ^+^ melanoblasts and regenerated melanocytes (melanin^+^) in the dorsum of *eartha* ^j23e1^ mutants (30.5 ± 9.2) is virtually identical to that in the wild-type larvae (31.0 ± 7.2). This result suggests that regenerated melanocytes in *eartha* ^j23e1^ larvae develop to late-stage (*dct* ^+^) but fail to produce melanin, and that *eartha* acts after the *dct* ^+^ stage in melanocyte differentiation during melanocyte regeneration (Figure 2A).

To test the specificity of *eartha^'^*s role in melanocyte regeneration, we asked if the *eartha* ^j23e1^ mutation affects other regeneration process, such as larval tail regeneration. When wild-type larval tails were amputated at 3 dpf, the wound first heals by closing the injured tip of the notochord and neural tube. This is followed by the outgrowth of fin fold in the ensuing two days. By 9 dpf (6 d postamputation), the missing larval tails are largely reconstituted, including complete regeneration of fin-fold tissues and melanocytes (Figure 2D and 2E). That most of these melanocytes are derived from undifferentiated precursors rather than immigration of previously differentiated melanocytes is demonstrated by the finding that larvae challenged to regenerate their tails in the presence of the tyrosinase inhibitor phenylthiourea (PTU) have few or no pigmented melanocytes (Figure 2F). Furthermore, the presence of newly differentiated but unpigmented melanocytes in these tails is revealed by the appearance of faintly pigmented melanocytes within a day after MoTP washout (Figure 2G) [29]. When similar tail amputation experiments were conducted in *eartha* ^j23e1^ larvae, their tails regenerated identically to those of wild-type larvae, except that we observed very few melanocytes in the regenerated tail. Those melanocytes that appear in the regenerated *eartha* tail were immediately distal to the amputation plane (Figure 2H and 2I), similar in position and numbers to those observed in wild-type regenerated tail in the presence of PTU. This result suggests that the immigration of the differentiated melanocytes may occur normally in *eartha* ^j23e1^ larvae, but that new melanocytes fail to regenerate. We interpret this result to suggest that *eartha* ^j23e1^ mutation specifically affects melanocyte regeneration but not general tail regeneration. In addition, the finding that the melanocyte regeneration defect also occurs in the tail regeneration experiments, where the small molecule MoTP is not used, indicates that the *eartha* phenotype is not a consequence of unanticipated interactions between MoTP and the mutated gene.

### *eartha* Mutation Affects Late-Stage Cartilage Differentiation

**Figure 3:**
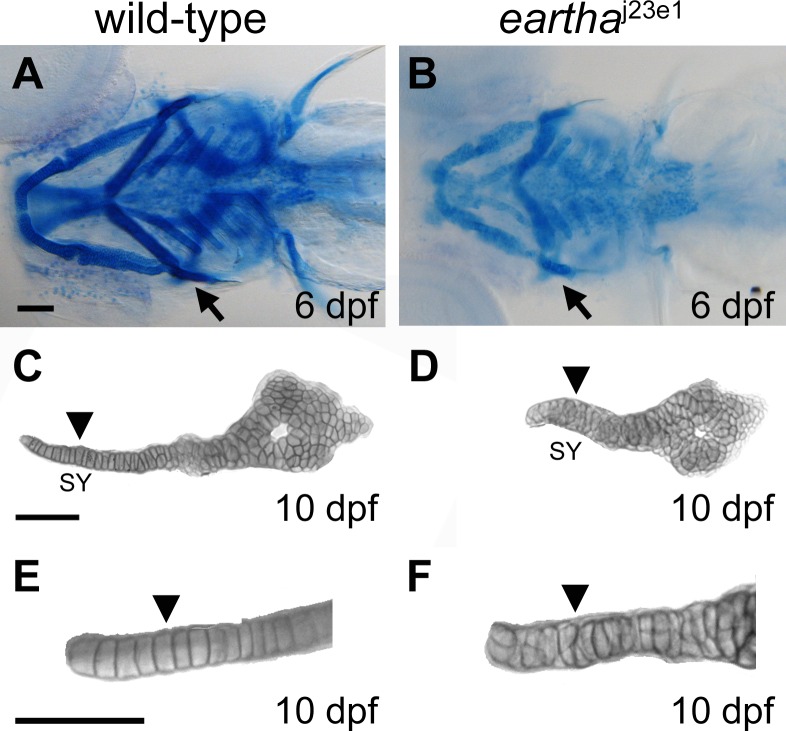
The *eartha* ^j23e1^ Mutation Affects Cartilage Maturation (A–F) The pharyngeal skeleton of wild-type and *eartha* ^j23e1^ larvae are revealed by Alcian Blue staining, where the staining in *eartha* ^j23e1^ larvae (B) is significantly weaker than that in wild-type larvae (A). At 10 dpf, the chondrocytes in the symplectic (SY) region (arrowhead in [C] and [E]) of the hyosymplectic cartilage are arranged into a single cell wide stack (arrowhead in [E]) in wild-type larvae. This organization of chondrocytes is disarranged in the *eartha* ^j23e1^ larvae (arrowheads in [D] and [F]). Arrows in (A) and (B) indicate the hyosymplectic cartilages that are shown in (C) and (D). Arrowheads in (C) and (D) mark the SY region of hyosymplectic cartilage. (E) and (F) are enlarged images of the SY region of hyosymplectic cartilage. Scale bars: 100 μm.

To further understand how *eartha* regulates developmental processes, we sought to examine the development of pharyngeal skeleton in *eartha* ^j23e1^ mutants. The pharyngeal skeleton develops from cranial neural crest cells. These arise from the dorsal part of the embryonic midbrain and hindbrain area, and migrate ventrally into the pharyngeal arches to form various cells and tissues, including cartilage. Mature cartilage includes chondrocytes and the surrounding ECM secreted by chondrocytes. At 4 or 5 dpf, the zebrafish pharyngeal skeleton and the cellular organization of chondrocytes can be robustly revealed by Alcian Blue staining, a dye that recognizes the cartilage matrix (Figure 3A). For instance, at this stage, chondrocytes in the symplectic (SY) region of the hyosymplectic cartilage are arranged into a single cell-wide stack, like a stack of coins (Figure 3C and 3E) [30,31]. *eartha* ^j23e1^ larvae develop shortened pharyngeal skeleton (Figures 1C and 3B). To assess the differentiation of the pharyngeal arches in *eartha* ^j23e1^ larvae, we use ISH with a variety of riboprobes, including *dlx2* [32] for migrating craniofacial neural crest and *sox9a* [33] and *type II collagen (col2a1)* [34] for differentiated chondrocytes. We found no differences in expression levels and patterns between the *eartha* ^j23e1^ and wild-type larvae for any of these markers above (unpublished data), indicating that chondrocytes for pharyngeal skeleton in *eartha* ^j23e1^ mutants are specified and then differentiate normally. In contrast, Alcian Blue staining, which marks cartilage, is significantly weaker in *eartha* ^j23e1^ larvae than in wild-type larvae (Figure 3A and 3B), suggesting a defect in cartilage maturation. In addition, we found *eartha* ^j23e1^ larvae develop all their pharyngeal cartilages, however the organization of chondrocytes is disarranged. For example, the chondrocytes in the SY cartilage fail to become arranged into the ordered cell stacks that are observed in wild-type larvae (Figure 3D and 3F). This disarrangement of chondrocytes causes the failure of pharyngeal skeleton extension into positions anterior to the eyes, accounting for the shortened head length (or hammerhead) phenotype. This observation that *eartha* ^j23e1^ mutants are defective in late-stage cartilage differentiation together with our analyses that indicate *eartha* mutation affects late-stage melanocyte differentiation during regeneration, may suggest that *eartha* plays a role in regulating late-stage cell differentiation.

### The eartha Phenotype Is Caused by a Nonsense Mutation in *gfpt1*

**Figure 4:**
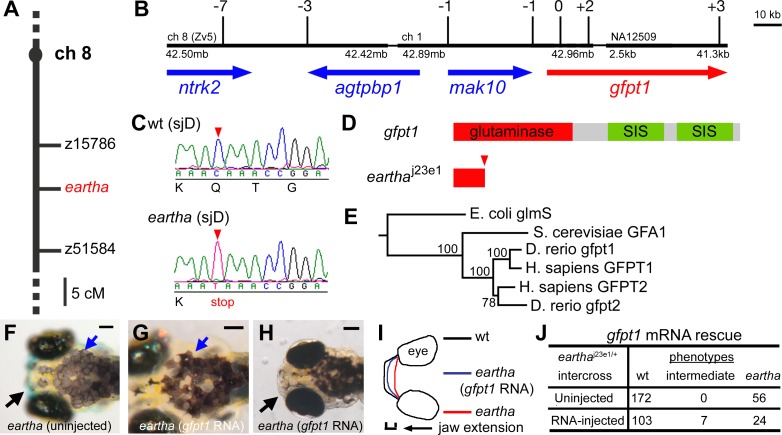
The *eartha* ^j23e1^ Mutation Is a Nonsense Mutation in *gfpt1* (A–E) The *eartha* ^j23e1^ mutation was mapped to Chromosome 8 between z15786 and z51584 (A). The physical map of the *eartha* locus was built from the zebrafish genome assembly version 5 (Zv5) with the support of recombination observed in 1,514 meioses. Shown here is a portion of this physical map delimited by seven recombinants at the proximal end and three recombinants at the distal end, containing approximately 189 kb comprising four genes: *ntrk2, agtpbp1, mak10,* and *gfpt1* (B). Sequencing of the *eartha* ^j23e1^ cDNA for *gfpt1* revealed a nonsense mutation in the third exon, where a CAA to TAA substitution resulting in Gln to stop cordon change (C) and (D). The phylogenetic tree of *gfpt* genes was constructed by using the neighbor-joining method with E. coli glmS as the out-group (E). (F–J) *gfpt1* mRNAs partially rescue the *eartha* phenotypes. *gfpt1* mRNAs were in vitro synthesized and injected into one to four cell stage embryos. At 5–7 dpf, 22% of the *eartha* ^j23e1^ larvae injected with *gfpt1* mRNA (J) have some distinctly darker melanocytes (blue arrows in [G]) and more extended pharyngeal skeletons (black arrow in [H] and [I]) than those in the noninjected *eartha* ^j23e1^ larvae (black and blue arrows in [F]). SIS: sugar isomerase domain. Scale bars: 100 μm.

**Figure 5:**
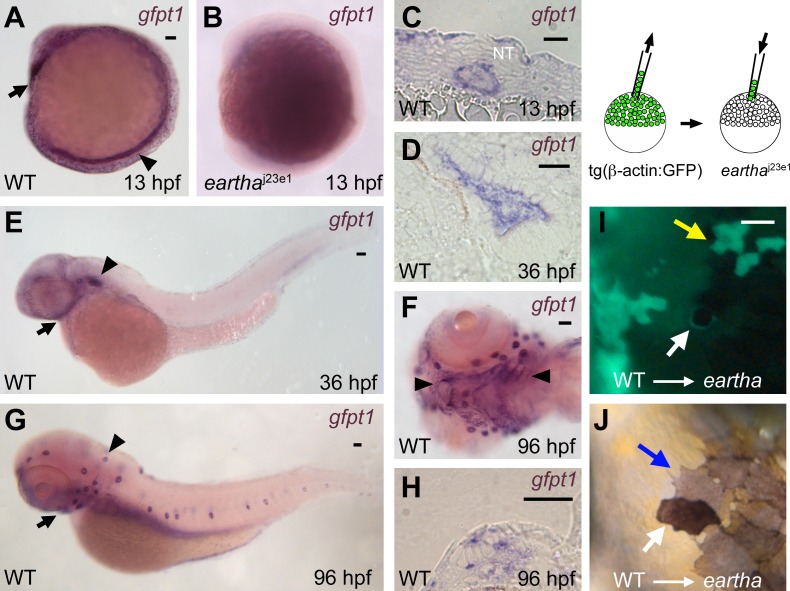
*gfpt1* Is Expressed in Notochord, Ear, and Pharyngeal Arches, and Acts Cell Autonomously in Melanocytes to Promote Melanocyte Darkening (A–H) ISH reveals high expression of *gfpt1* in the notochord (arrowhead in [A]) and the pillow (polster, arrow in [A]) at 13 hpf. No or very few *gfpt1* transcripts were detected in the *eartha* ^j23e1^ mutants at 13 hpf (B). A cross section (C) shows the *gfpt1* expression in notochord at 13 hpf. *gfpt1* is expressed in the ear (arrowhead in [E]) and pharyngeal arches (arrow in [E]) at 36 hpf. *gfpt1* expression in the sensory epithelium of the ear is shown in a cross section (D). At 96 hpf, *gfpt1* is expressed in the lateral line neuromasts (arrowhead in [G]) and the pharyngeal arches (arrow in [G] and arrowheads in [F]). A cross section (H) shows the *gfpt1* expression in the neuromasts. (I–J) Chimeric animals were generated by transplanting wild-type tg(β-actin:GPF) donor cells into *eartha* ^j23e1^ embryos at midblastula stage. A donor (GFP^+^) melanocyte in the *eartha* larvae is revealed following incubation in 0.1% epinephrine to induce melanosome contraction (white arrow in [I]). In the uncontracted state, the GFP^+^ (donor-derived) melanocyte appears darkly pigmented, in contrast to the lightly pigmented neighboring melanocytes (GFP^−^) that are derived from host (blue arrow in [J]). Yellow arrow in (I) indicates donor-derived skin cells (GFP^+^) that are not associated with darkened melanocytes. Scale bars: in (A), 50 μm for (A and B); in (C and D) and (H), 20 μm; in (E–G), 50 μm; in (I), 50 μm for (I and J).

To further understand how *eartha* regulates melanocyte regeneration, we took a positional cloning approach to identify the *eartha* ^j23e1^ mutation. We first mapped the *eartha* ^j23e1^ mutation to zebrafish Chromosome 8 by half-tetrad centromere linkage [35,36]. The *eartha* ^j23e1^ mutation was next mapped with a panel of 114 meioses to a 15 cM region between simple sequence repeat (SSR) markers z15786 and z51584 (Figure 4A). To further identify candidate genes, we built a physical map based on the sequences from the zebrafish genome assembly version 5 (Zv5, The Sanger Institute) by comparing sequence of the flanking markers z15786 and z51584 and several ESTs that were mapped within the region by radiation hybrid mapping (http://134.174.23.167/zonrhmapper/Maps.htm) [37]. Various assembly contigs were obtained and then bridged by additional fingerprinted BAC information (http://www.sanger.ac.uk/Projects/D_rerio/WebFPC/zebrafish/small.shtml). Finally, the accuracy of our physical map was tested and confirmed by mapping new SSR or single nucleotide polymorphism markers that were developed from each of the assembly sequences to a selected recombinant panel derived from 1,514 meioses (Figure 4B). By this fine mapping approach, a region containing the *eartha* ^j23e1^ mutation, flanked by seven recombinants at the proximal end and three recombinants at the distal end, was determined across approximately 189 kb comprising four genes: *ntrk2, agtpbp1, mak10,* and *gfpt1* (Figure 4B). A minimal critical region for the *eartha* ^j23e1^ mutation was further determined by a single proximal recombinant with a marker in *mak10* and two distal recombinants with a marker in the fourth exon of *gfpt1*. Sequencing of the cDNAs from the *eartha* ^j23e1^ mutants for candidate genes *agtpbp1* and *mak10,* which lie immediately outside the critical region, revealed no nucleotide difference from those cDNA derived from the originating nonmutagenized background (sjD strain). In contrast, we found a nonsense mutation in the third exon of *gfpt1* cDNA from *eartha* ^j23e1^ mutants (Figure 4C and 4D). This mutation is predicted to result in an unstable RNA transcript and a truncated protein. Consistent with this prediction, we seldom detected *gfpt1* RNA transcripts in the *eartha* ^j23e1^ mutants by ISH (Figure 5B).

To further test that *eartha* phenotypes result from loss of *gfpt1* function, we attempted to rescue *eartha* phenotypes by injecting *gfpt1* mRNA into larvae from *eartha* ^j23e1^/+ intercrosses. We observed that seven out of 134 larvae injected with *gfpt1* mRNA have intermediate or partially rescued jaw phenotypes at 7 dpf (Figure 4I and 4J), with jaws extending between 45–75 μm beyond their eyes. This amount of jaw extension falls between those observed in *eartha* larvae (21 ± 12.1 μm) and wild-type larvae (92 ± 17.1 μm) at the same stage (Figure 4H–4J). We also observed that five of these seven larvae show a mosaic pattern of dark (rescued) and light (unrescued) melanocytes (Figure 4G). Neither intermediate jaw length nor mosaic melanocyte pigmentation is observed in the uninjected larvae from a control *eartha* ^j23e1^/+ intercross (Figure 4F and 4J). In addition, genotyping with a closely linked SSR marker (see Materials and Methods) indicates that all of these partially rescued larvae are indeed homozygous *eartha* mutants. Taken together, with 24 unrescued *eartha* larvae, this result suggests that we partially rescued 22% (7/31) of the *eartha* mutants by injection with *gfpt1* mRNA. We note that, however, the mRNA injection experiments failed to rescue the much later melanocyte regeneration phenotype, presumably because the mRNA injected at cell stage 1–4 was no longer available at the late larval stages when melanocytes were challenged to regenerate. Taken together, the high-resolution mapping, cDNA sequencing, and mRNA rescue indicate that the *eartha* phenotype is caused by a null mutation in *gfpt1*.

*gfpt1* catalyzes the formation of glucosamine 6-phosphate and is the first and rate-limiting enzyme in this hexosamine biosynthesis pathway. In human, two related *GFPT* genes, *GFPT1* and *GFPT2*, have been described [38,39]. In the zebrafish Zv5 genome assembly, besides *gfpt1/eartha,* we identified a single additional *gfpt*-related gene. Phylogenetic analysis (Figure 4E) revealed that this additional gene is more closely related to the mammalian *glutamine:fructose-6-phosphate aminotransferase 2 (gfpt2)* gene than to zebrafish and mammalian *gfpt1;* therefore, we defined it as the zebrafish orthologue of *gfpt2*. The zebrafish *gfpt2* locus is located on Chromosome 21 (LG 21) near marker z28262 by our analysis of the zebrafish Zv5 genome assembly.

### Expression of *gfpt1* during Embryogenesis and Early Larval Development

To further explore how *gfpt1* expression and function may promote larval melanocyte regeneration following melanocyte ablation by MoTP, we examined the expression of *gfpt1* during early development. *gfpt1* transcripts were detected as early as the four-cell stage by reverse transcription-PCR and ISH and are distributed throughout the early embryo (unpublished data). ISH analysis demonstrated the restriction of *gfpt1* expression by 13 hpf, including intense expression in the notochord and the pillow (polster or future hatching gland) [40] at 13 hpf, otic vesicle and pharynx at 36 hpf, and lateral line neuromasts and pharyngeal arches at 96 hpf (Figure 5A–5H). We did not detect *gfpt1* transcripts in ontogenetic melanocytes or in regenerating melanocytes following MoTP washout. This failure to detect expression in melanocytes at these late stages could indicate that the relevant mRNA is transcribed at earlier embryonic stages when *gfpt1* expression in specific cells cannot be detected or that the abundance of *gfpt1* transcripts in melanocytes is below the threshold for detection. The alternative to the possibility that *gfpt1* acts autonomously in melanocytes is that *gfpt1* acts nonautonomously to the melanocytes to promote melanocyte differentiation, which is explored next.

### *gfpt1* Acts Cell Autonomously in Melanocytes to Promote Melanocyte Darkening

To test the cell autonomy of *gfpt1* for late-stage melanocyte differentiation, we took advantage of the ontogenetic defect of *eartha* mutants in melanocyte darkening, a late process in melanocyte differentiation. We generated chimeric larvae mosaic for *gfpt1* mutation by transplanting wild-type tg(β-actin:GFP) donor cells into *eartha* ^j23e1^ embryos at blastula stage (Figure 5I and 5J) [41]. We identified four *eartha* host larvae with a total of six GFP^+^ (donor-derived) melanocytes. In each case, the GFP^+^ melanocytes were also darkly pigmented, in contrast to the neighboring lightly pigmented melanocytes (GFP^−^) that are derived from host. Because the donor melanocytes show the wild-type phenotype (dark pigmentation) corresponding to their genotypes in the otherwise *eartha* ^j23e1^ mutation hosts, we conclude that *gfpt1* acts cell autonomously to promote melanocyte darkening.

We conclude that loss of *gfpt1* function results in a defect in melanocyte regeneration and that *gfpt1* acts at a late stage (after *dct* ^+^ stage) during melanocyte regeneration. Furthermore, chimera analysis shows that *gfpt1* acts cell autonomously to promote ontogenetic melanocyte darkening. These results lead to our inference that *gfpt1* acts cell autonomously in melanocytes to promote melanocyte differentiation from *dct* ^+^; melanin^−^ to melanin^+^ stage during melanocyte regeneration as well.

### *julie* Acts prior to dct Stage during Larval Melanocyte Regeneration

*julie* ^j24e1^ is the second mutation identified from our melanocyte ablation screen for defects specific to melanocyte regeneration. The development of *julie* ^j24e1^ embryos is indistinguishable from wild-type embryos until 3.5 dpf, at which time the size of their eyes and brain appears smaller than that of the wild-type larvae, although they have normal body length. We did not observed opaque or grainy cells that are typically reliable indicators of significant levels of cell death in zebrafish embryos, in the brains of *julie* ^j24e1^ larvae, suggesting that this small brain and eyes phenotype may result from growth retardation (Figures 1E and S1A and S1B). The notion that *julie* ^j24e1^ mutants have defects in growth rather than differentiation is further supported by our histological examination of eye structure in *julie* ^j24e1^ mutants that reveals normal retinal cell differentiation and lamination at 4 dpf despite the smaller size of their eyes (see Figure S1C and S1D). Most *julie* ^j24e1^ mutants die at approximately 7–8 dpf. Upon melanocyte ablation by MoTP, *julie* ^j24e1^ larvae regenerate 10.0% of the normal number of melanocytes compared to wild-type larvae at 5 dpf (Figure 1F and 1H). We also note that the few regenerated melanocytes in the dorsum of the *julie* ^j24e1^ mutants are distributed haphazardly throughout the length of the dorsum.

**Figure 6:**
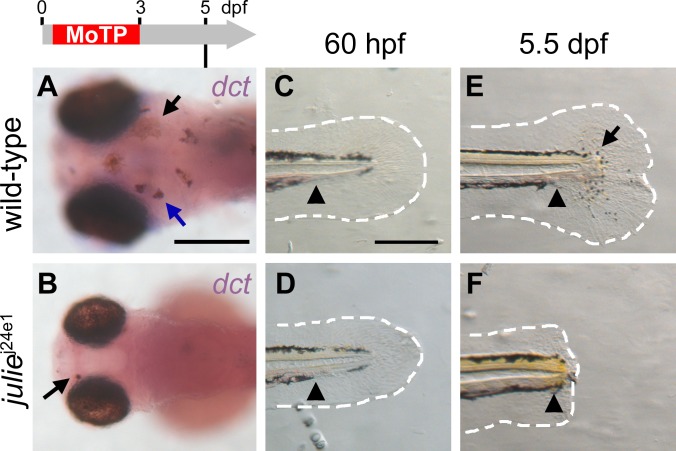
*julie* ^j24e1^ Mutation Disrupts Melanocyte Regeneration prior to the *dct* ^+^ Stage and Blocks Tail Regeneration Following Amputation (A and B) Larval melanocytes in *julie* ^j24e1^ and wild-type larvae were ablated by MoTP treatment from 14 to 72 hpf, and melanocyte regeneration was analyzed at 5 dpf by ISH with *dct* riboprobes for late-stage melanoblasts. At this stage, many *dct* ^+^ melanoblasts (melanin^−^, blue arrow in [A]) and melanocytes (melanin^+^, black arrow in [A]) appear in wild-type larvae (A). While few melanocytes (melanin^+^, black arrow in [B]) appear in the *julie* ^j24e1^ larvae, no *dct* ^+^ melanoblast was detected by ISH (B). (C–F) *julie* ^j24e1^ mutants fail to regenerate larval tails following amputation. Larval tails were amputated at 60 hpf (C and D) and tail regeneration in wild-type and *julie* ^j24e1^ larvae were examined at 5.5 dpf (E and F). At this stage, wild-type larval tails (E) were mostly reconstituted, including fin-fold tissues (outlined with white-dashed line) and melanocytes (arrow in [E]). In the *julie* ^j24e1^ larvae, the amputation wound healed but the *julie* ^j24e1^ mutant tail failed to regenerate (F). Arrowheads indicate the amputation planes in (C–F). The white-dashed lines outline the fin folds. Timeline (grey) above (A) indicates the period of MoTP treatment (red) and analysis time (vertical line below the timeline). Scale bars: 250 μm.

To understand how the *julie* ^j24e1^ mutation affects larval melanocyte regeneration, we examined melanocyte differentiation with the late-stage melanoblast marker *dct* in *julie* ^j24e1^ larvae after MoTP washout. We detected almost no *dct^+^* melanoblasts in *julie* ^j24e1^ larvae (Figure 6A and 6B), suggesting that *julie* ^j24e1^ mutation affects melanocyte regeneration prior to *dct* ^+^ stage.

To test whether *julie* has a specific role in melanocyte regeneration or whether it is also required for other types of postembryonic regeneration, such as larval tail regeneration (described above), we performed larval tail amputation on *julie* ^j24e1^ larvae. We found that the amputation wound heals in the ensuing few days after tail amputation, but that *julie* ^j24e1^ tails fail to regenerate (Figure 6C–6F). This defect together with the growth-deficient defect of the brain and eye suggests that *julie* may have a general role in postembryonic cell division or tissue growth.

### The *julie* ^j24e1^ Mutation Is a Splice-Site Mutation in *skiv2l2*

**Figure 7:**
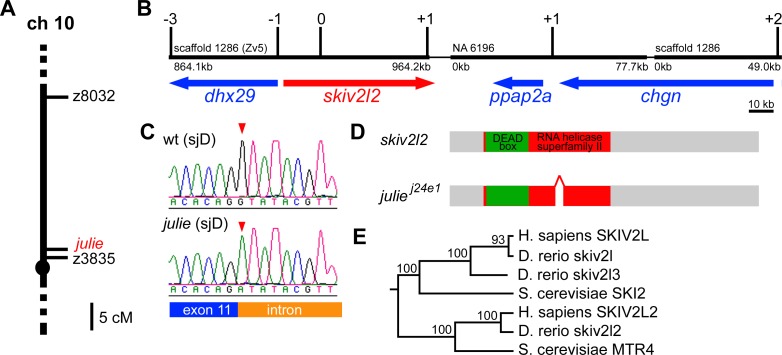
The *julie* ^j24e1^ Mutation Is a Splice-Site Mutation in *skiv2l2* (A) The *julie* ^j24e1^ mutation was mapped close to a marker z3825 on Chromosome 10. (B) The approximately 226.8 kb physical map of the *julie* locus was built from the zebrafish genome assembly version 5 (Zv5), flanked by three recombinants at the distal end and two recombinants at the proximal end from analyzing 438 meioses. A minimal critical region for the *julie* ^j24e1^ mutation was determined by a single recombinant with a distal marker in *dhx29* and one proximal recombinant with a marker in *skiv2l2*. (C) The genomic sequencing for *skiv2l2* locus in the *julie* ^j24e1^ mutants revealed a splicing site mutation (GT to AT change) at the 11th exon-intron boundary (red arrowheads). (D) Sequencing of *julie* ^j24e1^ mutant cDNA for *skiv2l2* revealed a 57 bp in-frame deletion at the 3′ end of exon 11 resulting in a partial deletion in the highly conversed DEAD-box domain in Skiv2l2 protein. (E) The phylogenetic tree of *skiv2l* genes was constructed using the neighbor-joining method with S. cerevisiae MTR4 as the out-group.

To further understand how *julie* regulates melanocyte regeneration, we sought to molecularly identify the *julie* ^j24e1^ mutation by a positional cloning method similar to the approach described above for the cloning of *eartha* ^j23e1^ mutation. We first mapped the *julie* ^j24e1^ mutation close to SSR marker z3825 on zebrafish Chromosome 10 (LG 10) by half-tetrad centromere linkage analysis (Figure 7A). Again, to build a physical map of the *julie* locus, we compared the sequence of the marker z3825 and several EST sequences that had previously been mapped to the region to identify various contigs from the zebrafish genome assembly version 5 (Zv5). These assembly contigs were then bridged by BAC sequences to generate the physical map shown in Figure 7B. The accuracy of this map was verified by mapping new SSR or single nucleotide polymorphism markers that were developed from the assembly sequences to a panel of selected recombinants derived from 438 meioses. The region containing the *julie* ^j24e1^ mutation, flanked by three recombinants at the distal end and two recombinants at the proximal end, comprises four genes, *dhx29, skiv2l2, ppap2a,* and *chgn*, stretching over approximately 226.8 kb (Figure 7B). A minimal critical region for the *julie* ^j24e1^ mutation was further determined by identifying a single distal recombinant with a marker within the *dhx29* gene and one proximal recombinant with a marker within the *skiv2l2* gene. Sequencing of *dhx29* revealed identical nucleotide sequences between cDNAs derived from *julie* ^j24e1^ mutant and that of the originating background (sjD strain). In contrast, by reverse transcription-PCR and sequencing analysis, we found a 57 bp in-frame deletion at the 3′ end of exon 11 in *skiv2l2* cDNA from *julie* ^j24e1^ mutants, which was not found in cDNA from sjD embryos. This deletion removes part of the conserved RNA helicase domain of *skiv2l2* (Figure 7D). This finding suggests a possible splicing-site mutation. Genomic sequencing of the *skiv2l2* locus further revealed a splice donor site mutation (GT to AT change) at the 11th exon-intron boundary in the *julie* ^j24e1^ mutants. This nucleotide change was not observed in the wild-type sjD strain on which the *julie* ^j24e1^ mutation was generated (Figure 7C and 7D). Taken together, our multiple lines of mapping results indicate that the *julie* ^j24e1^ mutation is a splice-site mutation that results in an in-frame deletion in the Skiv2l2 protein.

*skiv2l2* was thought to be closely related to *Saccharomyces cerevisiae SKI2* [24], a DEAD-box RNA helicase in a multiprotein nuclease complex, the exosome, which mediates 3′ to 5′ mRNA degradation in the cytoplasm [36,42]. By our phylogenetic analysis, we found that vertebrate *skiv2l2* genes are more closely related to yeast mRNA transport defective 4 *(MTR4)* gene (Figure 7E), an ATP-dependent DEAD-box RNA helicase that is thought to be involved in tRNA or rRNA degradation mediated by exosome in the nucleus [43]. Besides *skiv2l2*, we identified another two *superkiller viralicidic activity 2-like (skiv2l)*-related genes in the zebrafish Zv5 genome assembly. We defined a zebrafish *skiv2l* orthologue and a *skiv2l* related gene, *skiv2l3*. Our phylogenetic tree analysis shows that zebrafish *skiv2l3* is more closely related to vertebrate *Skiv2l* genes than to the *Skiv2l2*. This tree topology, together with our failure to find additional *skiv* homologues by BLAST analysis of the human or mouse genomes, suggests that the mammalian *Skiv2l3* gene was lost in the human lineage, but retained in that of zebrafish (Figure 7E).

### Expression of *skiv2l2* during Embryogenesis and Larval Development

**Figure 8:**
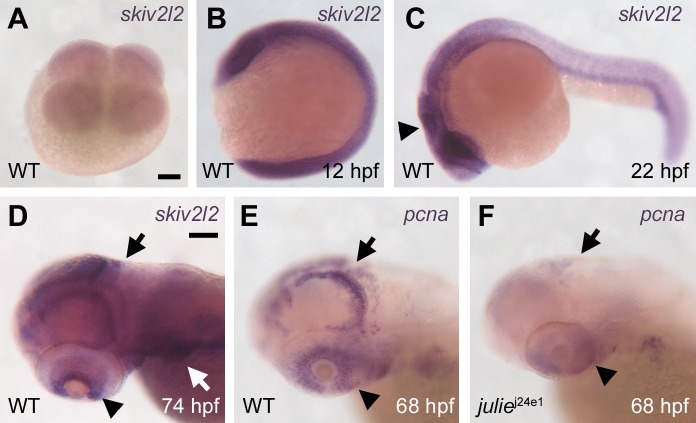
*skiv2l2* Expression Is Ubiquitous during Embryogenesis and Then Restricted to Proliferation Zones at Larval Stages (A and B) *skiv2l2* transcripts were detected as early as the four-cell stage by RNA ISH (A) and remained ubiquitously distributed in embryos throughout early embryogenesis (B) (12 hpf). (C and D) The expression of *skiv2l2* becomes stronger in the central nervous system (arrowhead in [C]) (22 hpf) and is then restricted to pectoral fins, cranial neural crest (white arrow in [D]), the medial and posterior parts of the tectum, and cerebellum (black arrow in [D]), and ciliary marginal zone of the retina (arrowhead in [D]) at larval stages (D) (74 hpf). (E) This larval-stage expression pattern of *skiv2l2* is nearly identical to the expression of *pcna* (68 hpf). (F) *pcna* expression is drastically reduced in *julie* ^j24e1^ mutants (68 hpf). Scale bars: in (A), 100 μm for (A–C); in (D), 100 μm for (D–F).

To understand how *skiv2l2* expression may regulate melanocyte regeneration, we examined the expression of *skiv2l2* during development by ISH. *skiv2l2* transcripts were detected as early as the four-cell stage (Figure 8A) and remained ubiquitously distributed in embryos throughout early embryogenesis (Figure 8B). The expression of *skiv2l2* becomes stronger in the central nervous system than the rest of the embryo after 16 hpf (Figure 8C) and is then restricted to pectoral fins, cranial neural crest, the medial and posterior parts of the tectum and cerebellum, and ciliary marginal zone of the retina at larval stages (Figure 8D). The expression of *skiv2l2* in these regions persists through 5 dpf (unpublished data). The medial and posterior parts of the tectum and cerebellum are proliferative zones during the brain growth at the larval stage, whereas the ciliary marginal zone is suggested to be populated with stem cells that contribute to retina growth throughout life in poikilotherms, such as amphibians and fish [44]. In addition, the expression pattern of *skiv2l2* is nearly identical to the expression of proliferating cell nuclear antigen *(pcna),* which marks proliferating cells and tissues at this larval stage (Figure 8E) [45]. To explore the possibility that *skiv2l2* promotes cell proliferation, we examined *pcna* expression in *julie* ^j24e1^ mutants and found that the expression of *pcna* is drastically reduced in these animals (Figure 8F), suggesting that *skiv2l2* is required for cell proliferation in these regions. This result is consistent with the phenotype of small eyes and brain observed in the *julie* ^j24e1^ animals, as well as the failure to regenerate melanocytes following melanocyte ablation by MoTP.

We sought to examine the expression of *skiv2l2* in regenerating melanocytes following MoTP washout. We were unable to detect any *skiv2l2* expression in possible melanoblasts or regenerating melanocytes at two to three days postMoTP washout (5–6 dpf) despite the strong detection of *skiv2l2* expression in various tissues described above. This failure could suggest the low abundance of *skiv2l2* transcripts, below our ability to detect by ISH, in regenerating melanocyte lineages.

Taken together, we suggest that loss of *skiv2l2* function causes the *julie* phenotype, and *skiv2l2* regulates cell division prior to the *dct* ^+^ stage during larval melanocyte regeneration.

## Discussion

We report a forward genetic screen aimed at identifying regulatory mechanisms specific to regeneration. We sought to isolate mutations specific to melanocyte regeneration by conducting a *F*_2_ parthenogenesis screen in zebrafish larvae employing a small molecule, MoTP, to ablate larval melanocytes. We specifically identified two inheritable mutations, *eartha* ^j23e1^ and *julie* ^j24e1^, which cause defects in larval melanocyte regeneration from our screen. Because this screen was far from saturation, these results indicate that many more mutant loci might be identified by further exploitation of this melanocyte ablation screen. Notably, these two mutants are also larval lethal with other defects in jaw development *(eartha)* or general larval growth *(julie)*. Thus, our findings suggest that many of the genes involved in regeneration processes are also parts of other developmental programs and that the number of genes required solely for regeneration may be small.

Because the mutations described here are late larval lethal, one possibility was that the melanocyte regeneration defects observed were a consequence of the impending death of the larvae. The finding that melanocyte regeneration occurs normally in *rapunzel* ^c14^ homozygous mutants, which die between 5 and 6 dpf, indicates that larval lethality is not a sufficient cause to the defects in melanocyte regeneration and instead indicates that the genes identified here contribute specific roles in regeneration. We also note that one of the mutations, *eartha,* blocks regeneration at a late stage of differentiation, suggesting that the regeneration process may differ from ontogenetic process not only in the as yet hypothetical mechanisms that keep stem cells in check or act to recruit them back into developmental pathways, but also in those later stages in differentiation that superficially seem to be held in common between ontogenetic and regeneration processes.

### *gfpt1* Promotes Melanocyte Differentiation from dct^+^; melanin^−^ to melanin^+^ Stage during Larval Melanocyte Regeneration

*eartha* ^j23e1^ mutation allows normal development of ontogenetic melanocytes (melanin^+^) but blocks larval melanocyte regeneration following MoTP treatment. We demonstrated that the phenotype of *eartha* ^j23e1^ mutants is caused by loss of *gfpt1* function. This conclusion is supported by several lines of evidence, including the high-resolution mapping of the *eartha* ^j23e1^ mutation, the identification of a nonsense mutation in the *gfpt1* locus, very few or no *gfpt1* transcripts detected in *eartha* ^j23e1^ mutants, and the partial rescue of the *eartha* ^j23e1^ phenotype by the injection of *gfpt1* mRNA. Our analysis of melanocyte differentiation during regeneration with the late-stage melanoblast marker *dct* reveals that a mutation in *gfpt1* blocks melanocyte differentiation after the *dct* ^+^ stage, but before they reach the melanin-positive stage. In addition, the finding that following larval tail amputation, *eartha* ^j23e1^ mutant larvae regenerate tails normally but fail to regenerate melanocytes in the tail indicates that *gfpt1* is not generally required for regeneration but instead identifies a mechanism specific to melanocyte regeneration.

*gfpt1* is the first and rate-limiting enzyme in the hexosamine biosynthesis pathway (glucosamine synthesis). The major end product of the hexosamine biosynthesis mediated by *gfpt1* is a sugar nucleotide UDP-GlcNAc, which is one of the building blocks for glycosyl side chains for glycoproteins, glycolipids, and proteoglycans [46]. Among these molecules, N-acetylglucosamine (GlcNAc) is found most frequently in the polysaccharide side chains of glycosaminoglycans (GAGs), a type of linear polysaccharide composed of repeated disaccharides [47]. For instance, hyaluronan, the simplest GAG, is made up of a repeated disaccharide unit, GlcNAc and glucurinic acid, and is part of the ECM in cartilages [48,49]. In addition to GAGs, GlcNAc is also found enriched in the N-linked or O-linked oligosaccharides (N-glycans or O-glycans), which attach to various intracellular glycoproteins, and serves as crucial modification in various developmental processes [50,51]. We note that in addition to contributing to polysaccharides, UDP-GlcNAc serves as an obligatory substrate for monosaccharide modification on serine or threonine residues of various proteins [52,53], and this monosaccharide modification, also referred to as O-linked GlcNAcylation, modifies proteins in a manner analogous to phosphorylation, and consequently regulates the cellular and developmental functions of these proteins [54]. Although in vitro biochemical studies showed that *gfpt1* regulates UDP-GlcNAc synthesis, whether *gfpt1* is the only enzyme for UDP-GlcNAc synthesis in vivo and whether other enzymes, such as *gfpt2*, also play roles in this process are unknown. Furthermore, the developmental role of *gfpt1* in vertebrates is unexplored.

The isolation and analyses of *eartha* ^j23e1^ mutants may provide insight into the roles of *gfpt1* in vertebrate development. Alcian Blue is a cationic dye that binds negatively charged sites on the GAGs, such as hyaluronan [55,56]. Since cartilage is largely composed of GAGs, Alcian Blue staining reveals the presence of these long chain sugar molecules. Our findings that chondrocytes in *eartha* ^j23e1^ mutants commit to their cell fates and develop to *col2a1* ^+^ stage, but display weaker Alcian Blue staining, suggest defects in formation of GAGs in the ECM of the cartilages. These results indicate possible defects in the ECM of the connective tissues and raise the possibility that the lightly pigmented phenotype of the ontogenetic melanocytes and the melanocyte regeneration defect we observed in the *eartha* ^j23e1^ mutants was also the consequence of defects in the ECM. Our finding by chimera analysis that wild-type or donor-derived melanocytes have normal pigmentation in the *eartha* ^j23e1^ mutant hosts indicates that this particular function is not mediated through ECM, but instead reflects a cell autonomous, presumably intracellular, function for *gfpt1* in melanocyte development. Although we have not yet explored the autonomy for *eartha* in melanocyte regeneration, it now seems likely that the role for *gfpt1* in melanocyte regeneration is autonomous as well.

Although the in vitro biochemical studies suggest a general role for *gfpt1* in the UDP-GlcNAc production, our analyses suggest that this pathway has only a handful of easily discerned and diverse consequences when disrupted in zebrafish embryos. Thus, the defect in jaw morphogenesis may indicate a role in regulating the proper formation of the ECM in chondrocyte development, while different roles in modulating intracellular targets may be revealed by the defects in melanocyte darkening during ontogeny or defects in progression from the *dct* ^+^; melanin^−^ to melanin^+^ stage during melanocyte regeneration.

One question remaining is how a mutation in *gfpt1* specifically affects the progression from *dct* ^+^; melanin^−^ stage to melanin^+^ stage in the regenerating melanocytes but has no similar effects on this stage of ontogenetic melanocyte development. One possibility is that the *gfpt1* mutation reveals a function specific to melanocyte regeneration that is not shared by embryonic melanocyte development. However, our observation that ontogenetic melanocytes in *eartha* ^j23e1^ mutants at 4–5 dpf have lighter pigmentation than those in wild-type larvae suggests that *gfpt1* also plays a role in embryonic melanocyte darkening. These findings may indicate that the two different phenotypes are due to the contribution of *gfpt1* from two distinct mechanisms, for instance that two distinct GlcNAc modified factors are responsible, respectively, for the two different stages/types of melanocyte development. The alternative possibility is that defects in a shared mechanism cause distinct phenotypes in ontogenetic and regeneration melanocytes. Differences between the roles in ontogenetic melanocyte development and larval melanocyte regeneration have been described for the *kit* receptor tyrosine kinase. Ontogenetic melanocytes differentiate in the absence of *kit* receptor tyrosine kinase function, but melanocytes in *kit* receptor tyrosine kinase mutants largely fail to differentiate when challenged to regenerate [9,17,57]. Therefore, the difference between ontogenetic and regeneration stages of melanocyte development in the *eartha* ^j23e1^ mutants may either reflect the effect of *gfpt1* on modification of a common substrate that in turn has important roles at different stages of melanocyte development or instead reflect two distinct pathways that are modulated by *gfpt1*'s role in UDP-GlcNAc production. Either of these models suggests some distinct mechanisms involved in regeneration that are not involved in ontogeny.

A less likely explanation that we cannot yet formally exclude is that the ability of ontogenetic melanocytes to reach melanin^+^ stage (light melanocytes) in the *eartha* ^j23e1^ mutants reflects the perdurance of *gfpt1* maternal message or protein. Such a model would suggest a maternal role in melanocyte differentiation at approximately 22–24 hpf when ontogenetic melanocytes begin to melanize. This stage of melanocyte development is far beyond the midblastula transition (4 hpf) [58,59], when zygotic transcription begins, and many maternal transcripts are degraded [60]. Furthermore, although we detected some maternal *gfpt1* transcripts in four-cell stage embryos, by 13 hpf, most or all *gfpt1* transcripts are derived from zygotic expression (Figure 5B), tending to argue against maternal perdurance. Moreover, morpholino oligonucleotides directed against the ribosome binding site of the *gfpt1* transcript (ATG blocking morpholinos), which might be expected to block the translation of maternal transcripts, cause embryonic melanocyte darkening defects similar to those observed in the *eartha* mutants, but no defect on the initial development and pigmentation of embryonic melanocytes (unpublished data). Although such results tend to argue against a role for maternal *gfpt1* transcripts in promoting embryonic melanocyte development, these experiments do not rule out the possibility of maternally supplied protein or other post-translational products being responsible for the embryonic melanocytes that are observed in the *eartha* mutants.

### *skiv2l2* Regulates Cell Division during Larval Melanocyte Regeneration

We identified a second mutant, *julie* ^j24e1^, which develops normal ontogenetic melanocytes but fails to regenerate melanocytes upon ablation by MoTP. The high-resolution mapping of the *julie* ^j24e1^ mutation reveals that *skiv2l2* is the best candidate in the *julie* ^j24e1^ critical region and excludes the possibility that the *julie* ^j24e1^ mutation is in the coding sequence of immediately flanking genes *dhx29* and *ppap2a* (Figure 6B). Furthermore, the identification of a splice-donor site mutation in the *skiv2l2* locus further supports the assertion that *skiv2l2* is the mutated gene in *julie* ^j24e1^ mutants. This splice-site mutation causes a 57 bp in-frame deletion in the helicase domain, which presumably leads to the disruption of the *skiv2l2* function [61].

Mammalian *skiv2l2* was originally thought to be closely related to yeast RNA helicase SKI2, which is known to be part of the cytoplasmic exosome complex mediating 3′ to 5′ mRNA degradation of selective mRNAs [62]. Our phylogenetic analysis (Figure 7E), however, reveals that vertebrate *skiv2l2*s are the orthologues for a different yeast RNA helicase MTR4. Yeast MTR4 was originally suggested to function as mRNA chaperones to unwind RNA duplexes and pack RNAs into stable structures for mRNA transport out of the nucleus [63,64]. More recently, MTR4 was found to be part of a nuclear complex TRAMP that polyadenylates noncoding RNAs to trigger their 3′ to 5′ degradation by the nuclear exosome complexes [65,66]. This exosome-mediated nuclear RNA degradation was proposed as a surveillance mechanism to control the quality and quantity of various nuclear RNAs, including rRNA, sn/snoRNA, and tRNA, and Mtr4p is a crucial factor for this degradation [43,65,67,68]. This mechanism is essential for 60S ribosomal unit biogenesis and also for yeast to grow [69]. However, the cellular function of *skiv2l2*, a vertebrate orthologue of MTR4, is unexplored.

The isolation of the *julie* ^j24e1^ mutation from our melanocyte ablation screen and our following characterization reported here may now shed light on the functional roles of *skiv2l2* in vertebrate development. Our finding of reduced *pcna* expressions in the proliferation zones of the eye and brain in the *julie* ^j24e1^ larvae suggests that *skiv2l2* plays a role in cell division. Furthermore, the finding of virtually no *dct* ^+^ melanoblasts in the *julie* ^j24e1^ mutants after MoTP washout suggests that *skiv2l2* may regulate melanocyte cell division at a relatively early stage (prior to *dct* ^+^ stage) during melanocyte regeneration. If the zebrafish *skiv2l2* has an equivalent biochemical function as that proposed for yeast MTR4 in transporting mRNA from the nucleus, it is possible that certain as yet unidentified mRNAs regulating cell proliferation may fail to be transported out of the nucleus in the *julie* ^j24e1^ mutants. This, consequently, would deplete their activities, thereby reducing cell proliferation in *julie* ^j24e1^ mutants. Alternatively, *skiv2l2* could function in the surveillance mechanism postulated in yeast to control the quality and quantity of various nuclear RNA species. This surveillance mechanism could be essential for proper ribosome assembly for protein translation, which is usually active in dividing cells. The defect in this surveillance mechanism could consequently lead to deficits in cell division in *julie* ^j24e1^ mutants. Finally, several DEAD-box RNA helicases in the exosome complex have been implicated in microRNA processing of transcripts for genes that subsequently regulate cell division, differentiation, and apoptosis during various aspects of organismal development including brain morphogenesis in zebrafish [70–73]. Whether *skiv2l2* plays a role in any of these aspects of RNA metabolism is an intriguing question for further investigation.

A remaining question is how a mutation in *skiv2l2* specifically affects melanocyte regeneration but not ontogenetic melanocyte development. One possible model is that *skiv2l2* specifically promotes cell division at postembryonic stages, including the cell division that is required for melanocyte regeneration. This possibility suggests a fundamental difference between the regulatory mechanisms used to generate the cells that differentiate to form the embryo and those that arise during larval growth. As discussed above, one explanation for this difference might simply reflect the role of perduring maternal products. Our finding that ATG blocking morpholino oligonucleotides against *skiv2l2* cause morphological defects similar to *julie* mutants (including smaller head and eyes) but still develop ontogenetic melanocytes argues against a role for maternally deposited transcripts accounting for ontogenetic melanocyte development observed in *julie* mutants (unpublished data). As we discussed for *eartha*, this does not rule out a role for maternally deposited Skiv2l2 proteins. A more interesting model is that the *julie* regeneration defect during the transition to larval growth may instead reflect the use of different types of precursors, which have different stem cell qualities of self renewal or multipotency than precursors for cells that differentiate during embryonic stages. Support for this notion of different types of precursors for embryogenesis and larval growth development may best come from the analysis of melanocyte development. Our previous work and that of others suggest that larval regeneration melanocytes and adult melanocytes are derived from self-renewal of precursors or stem cell [9,74,75]. In contrast, lineage analysis of zebrafish neural crest cells reveals little or no role for undifferentiated cells in the lineages that give rise to the embryonic melanocytes [76]. The requirement for *skiv2l2* at an early stage (prior to *dct* ^+^ stage) of melanocyte regeneration might then reflect a specific role in expanding the stem cell populations, or early steps in generating committed daughters from adult stem cells.

In summary, we isolated two mutations from our forward genetic screen and demonstrated their stage-specific roles for melanocyte regeneration. These mutations identify two mechanisms with specific roles in regeneration: *julie (skiv2l2),* which acts at an early stage and may promote postembryonic cell division and *eartha (gfpt1)*, which acts at a late stage and promotes melanocyte differentiation specifically from *dct* ^+^; melanin^−^ to melanin^+^ stage.

## Materials and Methods

### Fish stocks and rearing.

Wild-type fish stocks used for this manuscript were the sjC (C32) or AB inbred strain. The sjD inbred strain for mutagenesis was previously described by Rawls et al. [77]. All zebrafish were reared according to standard protocols at 28.5 °C [78], and their developmental staging in hpf and dpf, respectively, correspond to staging at the standard temperature of 28.5 °C [79]. All *rapunzel* ^c14^ mutants described here are homozygous and have been previously described [27]. We use two SSR markers to identify our mutation carriers from intercrosses: NA17592–1 (5′-TGCCTGACCAGCTGTTTCTA-3′; 5′-ACCAGCCTCCACTGAGATTC-3′) for *eartha* ^j23e1^ mutation and CR925725.7 (5′-CTGGCCCAAACCTACAAGTG-3′; 5′-TGTTGGACATATGACCCTTGA-3′) for *julie* ^j24e1^ mutation.

### Parthenogenesis screen.

Adult sjD inbred strain males were mutagenized by incubating them in 3 mM N-ethyl-N-nitrosourea (ENU), a chemical mutagen, for four one-hour periods over the course of six weeks [25,26]. The mutagenized sjD males were bred with sjC females to create a pool of *F*_1_ carriers with random mutations. Clutches of eggs from individual *F*_1_ females were collected and fertilized with UV-inactivated sperm. To exclude mutations that also cause defects in ontogenetic melanocyte development from our analysis, approximately ten to 20 of these fertilized eggs were set aside and allowed to develop into haploid embryos for the examination of their ontogenetic melanocyte development at 3 dpf [77]. The remaining fertilized eggs were subjected to an early pressure protocol to generate half-tetrad *F*_2_ diploid embryos [80] for the melanocyte regeneration assay. At 14 hpf stage, *F*_2_ diploid embryos were placed in 14 μg/ml MoTP for approximately a 58-hour incubation to ablate developing melanocytes, washed into egg water, then scored for melanocyte regeneration at 5–8 dpf [9].

### Whole-mount ISH.

ISH with antisense DIG-labeled riboprobes was performed as described [81], using 68 °C hybridization stringency washes, alkaline phosphatase-conjugated secondary antibodies, and NBT/BCIP (nitro blue tetrazolium chloride/5-Bromo-4-chloro-3-indolyl phosphate, toluidine salt) for color development (Roche, http://www.roche.com). Fish embryos were fixed in 4% paraformaldehyde (PFA). Probes for *dct* [82], *dlx2* [32], *sox9a* [33], and *col2a1* [34] were previously described. The full-length cDNAs of *gfpt1, skiv2l2,* and *pcna* were amplified from a cDNA pool of 3 dpf larvae and cloned into a pBluescript II vector (Strategene, http://www.stratagene.com). All the riboprobes were synthesized by T7 RNA polymerase (Promega, http://www.promega.com). To better visualize NBT/BCIP precipitates in otherwise dark mature melanocytes, some embryos and larvae were treated with 10 mM PTU to partially inhibit melanin synthesis. For ISH on larvae past 3 dpf, 1% DMSO was added to the 4% PFA to increase the tissue permeability, and riboprobes were fragmented to approximately 300 bp by alkaline hydrolysis [75]. For cryostat sections, embryos with ISH staining were fixed, embedded in 1.5% agarose, frozen in liquid nitrogen, and cut into 7–10 μm sections as previously described [78].

### Larval tail regeneration.

Tail amputations were performed on 3 dpf larvae with razor blades under a dissecting microscope. The tails were cut immediately posterior to the turn of caudal vessel, and amputated tissues included part of caudal tail fin fold, notochord, neural tube, and melanocytes. The tail regeneration was then assessed at 7–10 dpf (4–7 d postamputation).

### Alcian Blue staining.

To visualize the skeletal structures of the wild-type and *eartha* ^j23e1^ larvae, we stained the larvae with Alcian Blue (Sigma, http://www.sigmaaldrich.com), a dye that binds to the ECM associated with chondrocytes. Larvae at various stages were fixed at 4% PFA at room temperature for 24–48 h and then transferred into a solution of 0.1% Alcian Blue dissolved in 7:3 ethanol:glacial acetic acid. After 22 h staining, the larvae were rinsed with 7:3 ethanol:glacial acetic acid and subjected to a series of rehydration to distilled water. Tissues were cleared by incubating the larvae in prewarmed 0.05% trypsin dissolved in a saturated solution of sodium tetraborate for 1 h. The pigmentation of the larvae was bleached in a solution of 3% H_2_O_2_/1% KOH for one-hour incubation. The skeletal preparations were finally mounted in glycerol [28].

### Mapping.

The *eartha* ^j23e1^ and *julie* ^j24e1^ mutations were localized on LG 8 and LG 10, respectively, by half-tetrad centromere-linkage analysis [35,36] using SSR markers by early pressure treatment of *eartha* ^j23e1^/+^sjC^ and *julie* ^j24e1^/+^sjC^ oocytes. The sequences of SSR markers mapped to the vicinity of each mutation were searched against the zebrafish genome assembly version 5 (Zv5, http://www.ensembl.org/Danio_rerio) to identify sequenced contigs in each mutation region. We then linked these contigs by showing overlap with BAC ends or cDNAs. Additional SSR or SNP markers in our longer contigs were then developed and finely mapped to panels of mutant individuals, e.g., a panel of 1,516 mutant individuals generated from *eartha* ^j23e1^ / +^sjC^ intercrosses for *eartha* ^j23e1^ mapping or a panel containing 438 mutant individuals created from *julie* ^j24e1^/+^sjC^ intercrosses for *julie* ^j24e1^ mapping. The cDNAs of candidate genes were obtained by RT-PCR from 3 dpf homozygous mutants as well as from sjD and sjC wild-type animals, and sequenced for mutation identification.

### mRNA rescue.

The full-length cDNA of *gfpt1* was cloned into the pOX oocyte expression vector containing 5′ and 3′ *Xenopus* β-globin UTRs [83]. mRNAs were synthesized by T3 RNA polymerase (mMESSAGE mMACHINE, Ambion, http://www.ambion.com). We injected 40–100 pg of mRNAs with 1% phenol red into one to four cell stage embryos [84]. At 5–7 dpf, RNA-injected larvae were assessed for possible rescue phenotypes by measuring the jaw extension under Nikon SMZ1500 dissecting microscope. The genotypes of these larvae were then verified by the tightly linked SSR maker NA17592–1. We sought to rescue *julie* phenotype with the similar approach by *skiv2l2* mRNA injection. We observed no rescued *julie* larvae. This negative result is possibly due to the unavailability of the injected *skiv2l2* mRNA for postembryonic development.

### Phylogenic analysis.

The orthologuous sequences to *gfpt* and *skiv2l* genes were identified by BLAST search in the organism-specific genome database (http://www.ncbi.nlm.nih.gov/Genomes). Multiple sequences from various species were first aligned by ClustalW program (http://www.ebi.ac.uk/clustalw). The neighbor-joining method was used to construct the *gfpt* tree with Escherichia coli glmS as the out-group and the *skiv2l* tree with S. cerevisiae MTR4 as the out-group. Bootstrap values at all the branches for both trees were derived from 100 replicates using the maximum-likelihood method. The protein sequences of *Danio rerio ski2l* and *skiv2l3* are annotated from genomic sequence by using gene prediction method: GNOMON (http://www.ncbi.nlm.nih.gov/genome/guide/gnomon.html).

### Blastula transplantation.

To test the cell autonomy of the gene function, we generated chimeric animals by blastula transplantation [41]. Cell transplants were performed on embryos at the midblastula stage, for which 20–100 donor cells from wild-type tg(β-actin:GFP) embryos were injected into the host embryos from *eartha* ^j23e1^/+ intercrosses. *eartha* larvae with GFP mosaicism were collected and reared to the larval stage for cell autonomy analysis. To identify donor (GFP^+^) melanocytes in the *eartha* larvae, we first treated larvae with 0.1% epinephrine to contract their melanosomes for better GFP revelation and then examined the GFP expression in melanocytes under a dissecting microscope in the dark field. Once GFP^+^ melanocytes were found, their locations in the larvae were recorded by photography, and the larvae were then placed in fresh egg water with tricane, releasing the melanosome contraction, for the phenotypic assessment of those GFP^+^ melanocytes.

### Morpholino experiments.

Antisense morpholinos (MO) were designed and purchased from Gene Tools (http://www.gene-tools.com). Ribosome-blocking or ATG-blocking morpholinos to *gfpt1* (5′-GATACGCAAATATGCCACACATGTC-3′) or *skiv2l2* (5′-GTCCGCCATGTCTTCAAACAGACAC-3′) (1–25 pg) were injected into wild-type embryos at one-cell to four-cell stages and examined at 48 hpf for ontogenetic melanocyte development and at 3 dpf for melanocyte darkening in *gfpt1* MO-injected larvae and eye or head growth in *skiv2l2* MO-injected larvae [85].

## Supporting Information

Figure S1 Additional Phenotypes in *julie* ^j24e1^ Larvae (A and B) The melanocyte development in *julie* ^j24e1^ larvae is virtually identical to that in wild-type larvae. The size of brain and eyes of *julie* ^j24e1^ larvae (arrow in [B]) appears smaller than that of the wild-type larvae (arrow in [A]) at 3.5 dpf, although *julie* ^j24e1^ and wild-type larvae share the similar body lengths. No optically opaque or grainy cells, which are typically reliable indicators of significant levels of cell death in zebrafish embryos, are observed in the brains of *julie* ^j24e1^ larvae (arrowheads in [A and B]).
(C and D) Paraffin sections stained with hematoxylin (H) and eosion (E) show the normal retinal cell differentiation and lamination in the *julie* ^j24e1^ larvae (D) despite the smaller size of the eyes than that of wild-type (C). Scale bars: in (A), 250 μm for (A and B); in (C), 20 μm for (C and D).
(23 MB TIF)

### Accession Numbers

The GenBank (http://www.ncbi.nlm.nih.gov/Genbank) accession numbers for the genes described in this paper are *gfpt1* (DQ173928), *skiv2l2* (BC061456), *pcna* (BC064299), *dhx29* (XM_692841), *ppap2a* (XM_692415), and *chgn* (XM_001339600); *gfpt*-related gene (DQ173929, CR735104); *skiv2l* orthologue (XM_694641); and *skiv2l-*related gene, *skiv2l3* (XM_683014).

The GenBank accession numbers for protein sequences used in the phylogenetic analyses are as follows: E. coli glmS (NP_418185); S. cerevisiae GFA1 (NP_012818); D. rerio Gfpt1 (NP_001030153) and Gfpt2 (NP_001029093); and Homo sapiens GFPT1 (NP_002047) and GFPT2 (NP_005101); S. cerevisiae SKI2 (NP_013502) and MTR4 (NP_012485); D. rerio Skiv2l2 (BC061456), Ski2l (XM_694641), and Skiv2l3 (XM_683014); and H. sapiens SKIV2L2 (NP_056175) and SKIV2L (NP_008860).

## Abbreviations

*col2a1*: *type II collagen*
**dct**: *dopachrome tautomerase*
dpf: days postfertilization
ECM: extracellular matrix
EP: early-pressure
GAG: glycosaminoglycan
*gfpt1*: *glutamine:fructose-6-phosphate aminotransferase 1*
*gfpt2,*: *glutamine:fructose-6-phosphate aminotransferase 2*
GlcNAc: N-acetylglucosamine
hpf: hours postfertilization
ISH: in situ hybridization
MoTP: 4-(4-morpholinobutylthio)phenol
*MTR4*: *mRNA transport defective 4*
PTU: phenlthiourea
*skiv21*: *superkiller viralicidic activity 2-like*
*skiv212*: *superkiller viralicidic activity 2-like 2*
SSR: simple sequence repeat
UDP-GlcNAc: UDP-N-acetylglucosamine
